# Sleep duration in schooldays is associated with health-related quality of life in norwegian adolescents: a cross-sectional study

**DOI:** 10.1186/s12887-023-04306-5

**Published:** 2023-09-19

**Authors:** Erik Grasaas, Gudrun Rohde, Kristin Haraldstad, Sølvi Helseth, Milada Cvancarova Småstuen, Siv Skarstein, Hilde Timenes Mikkelsen

**Affiliations:** 1 Department of Health and Nursing Science, Faculty of Health and Sport Sciences, University in Agder, Kristiansand, Norway; 2 Department of Nursing and Health Promotion, Faculty of Health Sciences, Oslo Metropolitan University, Oslo, Norway; 3 Department of Clinical Research, Sorlandet Hospital, Kristiansand, Norway

**Keywords:** Adolescents, Sleep, Health-related quality of life, Self-efficacy, Mediation

## Abstract

**Background:**

Insufficient sleep is commonly reported in adolescence and is negatively associated with a wide range of health outcomes. A way to grasp the complicated challenge of how sleep impact different aspects of the adolescents´ everyday life is by examining the relationship between sleep duration in schooldays and weekends on different health-related quality of life (HRQOL) subscales. Furthermore, to expand the understanding of possible underlying mechanisms between sleep and HRQOL, testing for self-efficacy as a possible mediator is of importance.

**Methods:**

A cross-sectional study was performed among 696 adolescents aged 13–15 years from a school-based setting. All participants completed an electronic survey in their respective classrooms. The survey included demographic data, the School Sleep Habits Survey, the KIDSCREEN-27 questionnaire measuring HRQOL, and the General Perceived Self-Efficacy Scale. Statistical analyses were conducted using SPSS Statistics software including PROCESS macro by Andrew Hayes.

**Results:**

Our findings revealed overall similar sleep patterns in boys and girls including longer sleep duration in the study sample during weekends (10:09 h) than in schooldays (08:36 h). Regression analyses revealed that sleep duration in schooldays was positively and statistically associated with HRQOL subscales psychological well-being (95% CI [0.44 to 1.33]), autonomy and parents (95% CI [0.12 to 1.06]), school environment (95% CI [0.47 to 1.40]), and self-efficacy (95% CI [0.01 to 0.47]). Sleep duration in weekends revealed no associations with the HRQOL subscales, except for the HRQOL subscale psychological well-being (95% CI [0.09 to 0.85]). Mediation analyses revealed that sleep duration in schooldays explained most of the associations (64 − 75%) in 4 out 5 HRQOL subscales, except in the association with the HRQOL subscale physical well-being revealing an indirect effect of 71.9%.

**Conclusions:**

This cross-sectional study described sleep among Norwegian adolescents and demonstrated that sleep durations in weekends and schooldays impact HRQOL and self-efficacy, revealing overall better outcome in HRQOL and self-efficacy with sufficient sleep during schooldays. These findings support the regularity of sleep and highlight the importance of sufficient sleep during schooldays, especially in a school-based sample of adolescents.

## Background

Sleep is an essential factor for children’s and adolescents’ health and wellbeing [1]. Adolescence is a developmental period, wherein healthy sleep patterns often are neglected, and thus insufficient sleep is commonly reported in adolescence [2–4]. Further, adolescence is also an important period for mental, physical, and social development [5, 6]. Across countries and cultures, evidence suggests that adolescents do not get the recommended nine hours of sleep per night [7, 8]. Insufficient sleep in adolescence affects a wide range of health outcomes negatively [9–11]. Research evidence shows that the risk for physical, psycho-social and behavioral problems increases with insufficient sleep [10, 12–14]. Causes of insufficient sleep in adolescence are often divided into internal biological processes related to puberty including the shift in the circadian rhythm, and external factors such as excessive homework load, caffeine intake, less parental supervision of bedtime, perceived stress, later preferred sleeping time and evening use of electronic media [8, 10, 15–18].

Currently, there is extensive research investigating bedtime screen hours´ harmful effects on sleep and well-being [19, 20], such as the systematic literature review of Hale & Guan., which advised to limit or reduce screen time exposure in adolescence especially at bedtime [20]. In addition, Mireku et al., revealed consistent associations between night-time screen-based media device use and poor sleep outcomes and worse general health-related quality of life (HRQOL) [21]. With less sleep duration during the schooldays, adolescents have reported using the weekends for catch-up sleep [22]. In the systematic review of Sun et al., 72 studies were included to examine the associations of weekday-to-weekend sleep differences on health-related outcomes in school aged children and youths. Findings revealed that a larger discrepancy in weekday-to-weekend difference in bedtime was associated with depressive symptoms in youths, particularly in secondary school pupils. [23]. This review highlighting need for examine underlying mechanisms related to the impacts of weekday-to-weekend sleep duration [23]. A way to grasp the complicated challenge of sleep patterns impact adolescents´ everyday life is by examining the relationship between sleep duration in schooldays and weekends on different HRQOL subscales. HRQOL is a multidimensional construct that includes the individual’s subjective perspectives on the physical, psychological, social and functional aspects of health [24]. Due to the multidimensionality of HRQOL, it may provide essential insight into the diverse and complicated impacts of insufficient sleep in adolescents, and thus contribute to potentially mapping relevant aspects to improve [25]. Hence, HRQOL serves as a framework relevant to sleep, due to the positive focus on the individuals’ subjective perception of resources rather than problems [26].

As insufficient sleep affects different aspects of life [7, 10–17, 27, 28], which may over time result in fatigue, it is natural to assume that tiredness and fatigue affects a person’s beliefs in one’s capabilities (self-efficacy). Self-efficacy is a relevant intervening psychological variable when addressing health outcomes [29]. Albert Bandura defined self-efficacy as “one’s beliefs (cognition) in one’s capability to organize and execute the courses of action required to achieve given results” [30, 31]. Hence, self-efficacy might influence how one thinks, feels, motivates oneself, and behaves [32]. When addressing psychological variables, mediation analyses are often conducted and preferred, because a mediating variable transmits the effect of an independent variable on a dependent variable [33]. To expand the research field´s understanding of the relationship between sleep and HRQOL, possible mediators should be addressed to identify possible underlying mechanisms of the respective observed associations from an exploratory nature [34]. Research has shown that higher self-efficacy is positively associated with sleep, in students and adults [35–37]. Mikkelsen et al. reported that self-efficacy is associated with HRQOL among Norwegian adolescents [38]. Self-efficacy has been revealed as a mediator in the relationship between pain and HRQOL in different study samples of Norwegian adolescents [39, 40]. Moreover, self-efficacy is recently revealed as a mediator in the relationship between stress and HRQOL in Norwegian adolescents [41]. Still, the mediating role of self-efficacy on the relationship between sleep duration and HRQOL in Norwegian adolescents remains unknown. To inform practice and policy, and be able to develop health promoting interventions, it is important to expand the understanding of possible underlying mechanisms impacting the adolescents´ HRQOL. Testing for self-efficacy as a mediator in the relationship between sleep duration and HRQOL is thus of importance.

As sleeping habits among adolescents have changed in the last decades [3, 4], updated research evidence is needed to describe sleep in both schooldays and weekends of Norwegian adolescents. Further, exploring how their sleep duration in schooldays and in weekends may affect different aspects of HRQOL, which may provide new insight of their everyday life. Finally, to date little is known about self-efficacy as possible underlying mechanisms between sleep duration in schooldays and HRQOL in Norwegian adolescents. Hence, to address the current research gaps, the following aims are presented:

1. To describe sleep in Norwegian 13–15-year-old adolescents.

2. To explore possible associations between sleep duration in schooldays and weekends on HRQOL and self-efficacy.

3. Testing self-efficacy as a possible mediator on the relationship between sleep duration in schooldays and HRQOL.

We hypothesized that longer sleep duration in schooldays would reveal higher HRQOL and self-efficacy, and that self-efficacy would mediate the relationship between sleep duration and HRQOL.

## Methods

### Design

This cross-sectional study is a part of the “Start Young – quality of life and pain in generations” study, which is a Norwegian longitudinal study aimed at acquiring new knowledge about HRQOL and pain in adolescents and their parents [38]. The present study used baseline data collected from November 2018 to April 2019 collected for the specific purposes in “Start Young”, including this current paper.

### Study setting

A total of 59 schools covering ninth grade in elementary school from the south-eastern part of Norway were invited to participate. Twenty-two schools agreed to take part. The schools varied in localization (from city to suburb) and size, admitting adolescents with different economic and sociocultural backgrounds. Potential participants consisted of 1663 adolescents in the ninth grade (students aged 14–15 years) from the participating schools, of whom 696 participated and answered the questionnaire (response rate, 41.8%). The response rate varied across schools from 8.6 to 92.1%.

### Study procedures

Approximately one week before the data collection, project members visited the participating schools to provide the adolescents with verbal and written information about the study. Parents received written information. Informed consent was obtained from all the participants and parents/legal guardians for the study. A web-based questionnaire was administered to the adolescents and completed inside the classrooms during school hours. A teacher and one or two project members were present to aid when needed. All collected data was stored in a safe data server at the University of Oslo, Services for sensitive data (TSD), which is in compliance with Norwegian legislation for collecting, analysing and storing sensitive research data [42]. All study procedures were approved by the Norwegian Centre for Research Data (Reference number: 60.981).

### Measures

#### Demographic variables

The first part of the questionnaire included questions regarding demographic data, such as gender, age and living conditions. Moreover, the questionnaire included the following study variables: sleep (independent variable), HRQOL (dependent variable), and self-efficacy (mediator variable).

### Questionnaires

#### Sleep

Sleep was assessed using selected questions adapted from the Norwegian version of the School Sleep Habits Survey [43]. We used questions about usual sleeping and waking behaviors in schooldays and weekends over the past two weeks, and questions about daytime sleepiness and the feeling of getting a sufficient amount of sleep. The survey has an established validity in comparison to actigraphy and sleep diaries and has been widely used for adolescents [44], including Norwegian adolescents [38]. The time for going to bed and time for wakening was recoded into numeric order for providing accurate estimates of mean and standard deviation (SD) for sleep duration in schooldays and weekends. Thus, sleep duration in schooldays and weekends was respectively computed by subtracting the difference in hours between time of wakening and time for going to bed. The decimals were converted into minutes simply by multiplying with 60.

#### HRQOL

HRQOL was assessed using the Norwegian version of the KIDSCREEN-27 questionnaire [45]. This questionnaire is organized into the following five subscales: (1) physical well-being, (2) psychological well-being, (3) autonomy and parents, (4) social support and peers, and (5) school environment, and is considered a valid and reliable multidimensional measure of HRQOL in adolescents [26, 46–48]. KIDSCREEN-27 comprises a 5-point rating scale (e.g., from “never” to “always”). We computed Rasch-scores and transformed them into *t*-values that are normed to a mean of 50 and a standard deviation (SD) of 10 [49]. Higher values in the respective subscales indicate better HRQOL and well-being. All subscales showed satisfactory Cronbach’s alpha values above 0.7 in the present study.

### Self-efficacy

Self-efficacy was assessed using the Norwegian 10-item version of the General Perceived Self-Efficacy Scale [50]. This scale is developed to identify a person’s optimistic self-belief and global confidence in one’s abilities to cope with the tasks, demands and challenges of life in general, and is considered a valid and reliable psychometric scale, also in adolescents [51–53]. It comprises 10 statements that the respondent rates on a scale from 1 (completely wrong) to 4 (completely right). The individual scores are then summed and divided by 10 to obtain a self-efficacy score ranging from 1 to 4, wherein higher scores indicate higher levels of self-efficacy. The subscale showed a satisfactory Cronbach’s alpha value of 0.87 in the present study.

### Statistical analyses

The statistical analyses were conducted using IBM SPSS Statistics for Windows, Version 25.0 (IBM Corp., Armonk, NY, USA). Descriptive measures were used for the sociodemographic data. Continuous variables were described using mean and standard deviation, and categorical variables with frequencies and percentages. Linear regression analyses were conducted between the independent variable sleep duration in schooldays and in weekends on the dependent variables HRQOL subscales and self-efficacy.

**Fig. 1:**
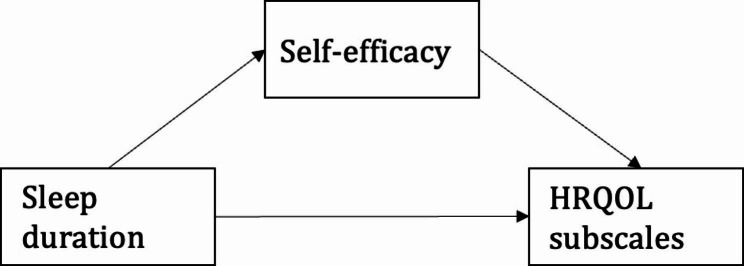
Illustration of the simple mediation model

Prior to the mediation analyses, statistical considerations were addressed. Based on the respective findings of the linear associations and the scope of the paper with corresponding hypotheses, sleep duration during schooldays was selected as the most relevant independent variable on HRQOL for the mediation analysis. Further, as the precondition of a significant relation between the dependent and independent variable is not needed for proceeding with the mediation analyses [54], all HRQOL subscales were included in the mediation analysis. Moreover, due to the homogeneity of the sample, controlling for variables such as gender and age were not considered appropriate for the mediation model. To assess the indirect effect of self-efficacy, mediation analyses were conducted using the PROCESS macro model 4 by Andrew Hayes [55]. The estimates were calculated using bootstrapping to increase precision and the mediation effect was regarded as statistically significant if the 95% confidence interval (CI) for this effect did not include 0. To present the mediation findings in percentages, both the indirect and direct effects were each divided by the total effect, multiplied by 100. *p*-values < 0.05 were considered statistically significant and all tests were two-sided. We used a simple mediation model presented in Fig. 1.

## Results

### Participants

In total, 696 Norwegian adolescents participated in this study, 57.5% were girls and 42.5% were boys. The mean age of the participants was 14.1 years (SD, 0.33), ranging from 13 to 15 years. Most adolescents were living at home together with two parents (73.0%), some adolescents switched between living with their mother and father (14.4%), other lived together with one parent (7.9%) or one parent with stepdad/-mother (2.9%) or in other arrangements (1.9%).

### Descriptive data of sleep patterns in norwegian adolescents

The adolescents were on average going to bed at 22:22 (SD, 50 min) on schooldays, whereas there were only minor differences between girls 22.19 (SD, 50 min) and boys 22.25 (SD, 51 min). Girls reported a time of wakening at 06:56 (SD, 43 min) in schooldays and boys at 07:00 (SD, 51 min), revealing close to no difference in average sleep duration in schooldays for girls (8.37 h) and boys (8.35 h). The sleep duration during weekends (10.09 h) were overall higher than in schooldays and similar in girls and boys. The majority of the sample (57%) reported that they never woke up during the night (Table 1). About 40% of the study sample reported that they never felt sleepy during daytime activities, whereas this was reported by 32% of the boys and 51% of the girls. There were close to no difference between girls and boys when combining “not at all reported sleepy” or “a little sleepy” (90.2% versus 91.5%, respectively). About two thirds of the sample reported that they always or usually get enough sleep.

**Table 1:** Characteristics of sleep patterns for the study sample

Study variable	All (n = 696) (mean/SD)	Girls (n = 400) (mean/SD)	Boys (n = 296) (mean/SD)
Going to bed in schooldays (hours/min) ^a^	22:22 (0.50)	22:19 (0.50)	22:25 (0.51)
Time of wakening in schooldays (hours/min)	06:58 (1.00)	06:56 (0.43)	07:00 (1.23)
Sleep duration in schooldays (hours/min) ^b^	8.36 (0.52)	8.37 (0.58)	8.35 (1.48)
Going to bed during weekends (hours/min) ^a^	23:59 (1.17)	23:56 (1.16)	00:04 (1.22)
Time of wakening in weekend (hours/min)	10:08 (1.35)	10:06 (1.19)	10:10 (1.35)
Sleep duration in weekend (hours/min) ^c^	10.09 (1.39)	10.10 (1.46)	10.06 (1.24)
Times of wakening during a night (N %)			
Never Once Two or three times More often Not sure	401 (57.7) 188 (27.1) 57 (8.2) 8 (1.2) 4 (5.9)	214 (53.6) 117 (29.3) 43 (10.8) 5 (1.3) 20 (5.0)	187 (63.2) 71 (24.0) 14 (4.7) 3 (1.0) 21 (7.1)
Feeling sleepy during daytime activities (N %)			
Not at all A little bit More than a little A big problem A very big problem	280 (40.3) 311 (44.7) 68 (9.8) 26 (3.7) 10 (1.4)	129 (32.3) 191 (47.9) 51 (12.8) 19 (4.8) 9 (2.3)	151 (51.0) 120 (40.5) 17 (5.7) 7 (2.4) 1 (0.3)
Feeling of sufficient sleep (N%)			
Always Usually Sometimes Rarely Never	59 (8.5) 387 (55.7) 177 (25.5) 63 (9.1) 9 (1.3)	25 (6.3) 212 (53.1) 108 (27.1) 48 (12.0) 6 (1.5)	34 (11.5) 175 (59.1) 69 (23.3) 15 (5.1) 3 (1.0)

^**a**^ The variable was recoded into numeric order after midnight for estimating mean and SD

^b^ The variable was computed by subtracting the numeric wakening time and time for going to bed in schooldays

^c^ The variable was computed by subtracting the numeric wakening time and time for going to bed in weekends

### Associations between sleep duration in schooldays and weekends on HRQOL subscales, and self-efficacy

Longer sleep duration in schooldays revealed higher HRQOL scores in the subscales psychological well-being, autonomy and parents, school environment and for self-efficacy, all statistically significantly associations (*p* < 0.05). The two largest regression coefficients were revealed between sleep duration in schooldays and the HRQOL subscales psychological well-being (B = 0.88) and school environment (B = 0.93). Sleep duration in weekends revealed no associations with the HRQOL subscales or self-efficacy (Table 2), except for the HRQOL subscale psychological well-being (B = 0.47, *p* < 0.05).

**Table 2:** Linear regressions of sleep duration in schooldays and weekends (independent variables) on the HRQOL subscales (dependent variables) and self-efficacy (dependent variable)

Study variable	mean/SD	Sleep duration in schooldays			Sleep duration in weekends		
		B	95% CI	*p-*value	B	95% CI	*p-*value
Physical well-being	47.08 (9.34)	0.32	-0.18 to 0.82	0.20	0.13	-0.29 to 0.55	0.55
Psychological well-being	46.61 (8.42)	0.88	0.44 to 1.33	< 0.01	0.47	0.09 to 0.85	0.02
Autonomy and parents	52.60 (8.76)	0.58	0.12 to 1.06	0.01	0.22	-0.18 to 0.62	0.28
Social support and peers	48.44 (8.47)	0.43	−0.03 to 0.88	0.07	0.27	-0.12 to 0.65	0.17
School environment	48.04 (8.57)	0.93	0.47 to 1.40	< 0.01	0.09	-0.30 to 0.48	0.64
Self-efficacy	31.08 (4.31)	0.24	0.01 to 0.47	0.04	0.11	-0.08 to 0.31	0.25

B, unstandardized coefficient, CI Confidence interval

### Mediation effect of self-efficacy on the relationship between sleep duration in schooldays and HRQOL subscales

**Fig. 2:**
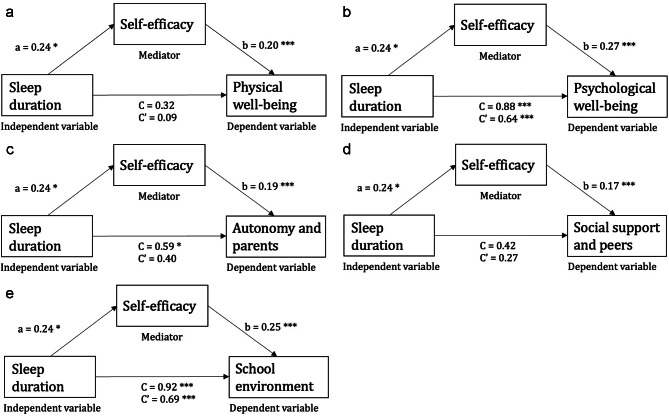
Mediation effect of self-efficacy on the association between sleep duration in schooldays on HRQOL subscales (physical well-being, psychological well-being, autonomy and parents, social support and peers, and school environment). *p < 0.05, **p < 0.01, ***p < 0.001

The mediation analyses revealed significant indirect effects of self-efficacy on all HRQOL subscales; physical well-being (95% CI [0.05 to 0.59]), psychological well-being (95% CI [0.04 to 0.66]), autonomy and parents (95% CI [0.02 to 0.50]), social support and peers (95% CI [0.00 to 0.05]), and school environment (95% CI [0.03 to 0.62]). The estimates of regression coefficients are illustrated in Fig. 2 as path a and path b. Path C represent the total effect of the association between the independent variable and the dependent variable (including the indirect effect), whereas C′ represent the direct effect of the independent variable on the respective HRQOL (excluding the indirect effect).

Sleep duration in schooldays (C′) explained the majority of the total path in 4 out of 5 HRQOL subscales, with a direct effect ranging from 64.3 to 75.0%. The highest mediating effect was revealed in the HRQOL subscale physical well-being revealing an indirect effect of 71.9% (Table 3). The lowest indirect effect (25.0%) was found for the HRQOL subscale school environment.

**Table 3:** Direct effects of sleep duration in schooldays and indirect effects of self-efficacy presented as proportions (percentages) of the total effect on HRQOL subscales

HRQOL subscale	Direct effect (%)	Indirect effect (%)
Physical well-being	28.1	71.9
Psychological well-being	72.7	27.3
Autonomy and parents	67.8	32.2
Social support and peers	64.3	35.7
School environment	75.0	25.0

## Discussion

In this study we aimed at describing sleep in Norwegian 13–15-year-old adolescents, explore possible associations between sleep duration in schooldays and weekends on HRQOL and to test self-efficacy as a possible mediator on the relationship between sleep duration in schooldays and HRQOL. Our descriptive findings revealed that sleep duration during weekends were overall higher than in schooldays for both genders. Sleep duration in schooldays was positively associated with higher HRQOL for the subscales psychological well-being, autonomy and parents, school environment and self-efficacy. Sleep duration in weekends was positively associated with higher levels of HRQOL for the subscale psychological well-being. In the association between sleep duration in school days and HRQOL subscales, mediation analyses revealed that sleep duration explained most of the associations in 4 out of 5 HRQOL subscales, except in the association with the HRQOL subscale physical well-being revealing an indirect effect of 71.9%.

Our descriptive findings revealed similar findings in boys and girls in terms the time of going to bed and time of wakening, revealed close to no gender difference in the total sleep duration in schooldays and weekends. Moreover, our study sample reported a sleep duration with over eight and a half hours of sleep in schooldays and over ten hours in weekends, which is close to be in accordance with the recommended nine hours of sleep per night [7, 8]. However, methodological aspects should be discussed, as the administered sleep questionnaire in this current study ask when the adolescents are going to bed, not the specific sleep onset time (SOT). Thus, probably creating an overestimation of sleep duration, compared to other international studies [10, 17, 56]. On the other hand, about two thirds of our study sample reported that they always or usually get enough sleep. Still, findings in Norway by Saxvig et al., showed that 84.8% of older adolescents aged 16–17 years failed to obtain the recommended amount of sleep (8 + hours) on schooldays [57]. Moreover, Saxvig and colleagues discussed and highlighted the discrepancies in findings between time in bed and actual sleep duration on schooldays. These findings are of importance, as they reveal how sleep duration may be overestimated by only using time in bed as an indicator in adolescents [57]. Nevertheless, time spent in bed as an indicator of sleep duration is still an interesting finding among adolescents, and several previous studies, presented in the review and metanalysis by Gardisar et al., do not report SOT and thus, might also overestimate sleep duration [17]. When SOT are not reported, research evidence suggests that SOT is on average 16.8 min later than the time of going to bed for adolescents aged 15–18 [58].

An interesting finding from the descriptive analyses is the one-and-a-half-hour discrepancy in sleep duration between schooldays and weekends, which is in accordance with American teenagers in secondary school [59]. Weekday-to-weekend sleep discrepancy is commonly reported in adolescents in school-age [23] and often peaks in late adolescence and early adulthood with a discrepancy of up to 3 h [60]. This discrepancy should not be neglected as the systematic review by Sun et al., shows clear trends. A large discrepancy in weekday-to-weekend difference in adolescents, especially in secondary school, is associated with poorer academic performance, depressive symptoms, higher risk of substance and higher risk of overweight and obesity [23]. Increased weekend catch-up sleep is reported as an indicator of insufficient sleep in schooldays and thus associated with poorer performance in adolescents on attention tasks [22]. The sleep timing between schooldays and weekends is often referred to as a “social jetlag” as their sleep schedule tends to have less obligated tasks during weekends compared to schooldays, and thus both the time of going to bed and time of wakening is affected.

Our regression analyses of sleep duration on HRQOL subscales and self-efficacy in schooldays and in weekends demonstrated all positive coefficients, which presumably indicates and supports the importance of sleep duration on adolescents’ wellbeing. These findings are in accordance with the literature, clearly emphasizing the importance of sufficient sleep on health outcomes in adolescents [9–11, 14, 15]. When assessing our current findings further, a significant association was found between sleep duration in schooldays and self-efficacy, but not in the association between sleep duration in weekends and self-efficacy, which is interesting. Since, higher degree of self-efficacy in adolescents have been reported to positively influence academic performance, the likelihood of remaining in school and is associated with higher HRQOL [61–63]. In students, higher self-efficacy is reported to predict better sleep quality [37] and positively influence the time of falling asleep after bedtime [64], thus multiple directions of associations seems to be of relevance as self-efficacy may both predict sleep and may also be influenced by sleep. These findings support our hypothesis of addressing self-efficacy as a mediator in the relationship by assessing the underlying mechanisms in the respective study variables. Further, sleep duration in schooldays was associated with HRQOL subscales such as psychological well-being, autonomy and parents, and school environment, which presumably indicate how sleep duration in schooldays impact several aspects of life among adolescents throughout their everyday life, including their perceptions of school, psychological well-being, and home with parents. Further, it is interesting to discuss the associations between sleep duration in schooldays and sleep duration in weekends on the HRQOL subscale autonomy and parents, due to the respective nonsignificant findings in weekends. These findings might indicate why sufficient sleep in schooldays are of importance. The demand in schooldays differ compared to weekends for most adolescents. During schooldays there is a need for higher degree of autonomy, ability to structure homework, home duties, interactions with parents, social responsibilities, and other activities. Sufficient sleep in schooldays might therefore be a key element for being able to grasp these demands. In weekends, adolescents often experience a time with higher degree of freedom and thereby sleep duration seems to have a lesser impact on their relation to parents and degree of autonomy.

The mediation analysis revealed significant indirect effects of self-efficacy on the relationship between sleep duration in schooldays and all HRQOL subscales, as hypothesized. Although, we found significant indirect effects of self-efficacy on the relationship between sleep duration in schooldays and respective HRQOL subscales, the increase of HRQOL in 4 out of 5 subscales was primarily explained by the adolescents´ duration of sleep in schooldays rather than self-efficacy. The lowest indirect effect (25.0%) was found for the HRQOL subscale school environment, which is interesting, because it reveals that sleep duration is of importance, as it explains the majority of the total path of the relationship. However, for the HRQOL subscale physical well-being the majority of the path was explained by the indirect effects of self-efficacy, which is in accordance with earlier findings among Norwegian adolescents in the relationship between persistent pain and HRQOL subscales, wherein similar high degree of self-efficacy was shown in the subscale physical wellbeing [39]. Self-efficacy is a determinant for physical activity and thus seems logical that self-efficacy primarily explains the degree of the association. It is likely to assume that adolescents with insufficient sleep, may feel fatigued, which again affect their confidence in the ability to perform a given task (self-efficacy), especially a physical task. The construct of the physical well-being dimension administered in this current study provides a subjective perception of the adolescents´ physical well-being, however the respective questions are related to physical tasks, which may help explain the high indirect effect of self-efficacy in the association between sleep duration in schooldays and the HRQOL subscale physical well-being. These findings may be one of several underlying mechanisms explaining the findings in a recent systematic review, showing longer periods of sleep in adolescents is associated with higher levels of physical fitness [65]. Self-efficacy is also reported to have positive association to quality of life in adolescents [38, 66, 67], and as it is considered as a changeable concept [68], it therefore seems like a potential concept to intervene upon when designing interventions aiming to promote HRQOL and sleep in adolescents.

### Strengths and limitations

Several strengths and limitations should be considered when interpreting the current findings. Firstly, our estimation of sleep duration is based on the adolescents´ time of going to bed and time of wakening. This should be considered as major limitation as we do not possess data of SOT, which probably have influenced the accuracy in the statistical estimates and resulted in an overestimation of sleep duration and thus reduced the generalizability of findings (i.e., external validity). In this cross-sectional data, no causal interactions can be determined. We built our mediation model on assumptions of current research evidence and based on our understanding of the research field but have no guarantee that our assumptions are correct. Still, by exploring cross-sectional data using the mediation model, the understanding of potential underlying mechanisms can be revealed, which is important to address for further expanding the research field. We believe it is a strength that all HRQOL subscales were included in the meditation analyses, regardless of their level of significance of the associations in the linear regressions, as it contributed to reveal important findings of the indirect effect of HRQOL subscale physical well-being. In this current study sample, we do not possess information of their parents´ educational background, income, or work status, which could be considered appropriate confounders to address. Aspects such as possible medical and social confounders were not possible to adjust for and should be considered as a limitation as they could affect sleep duration. In addition, controlling for sex did not reveal any changes in the significance of our results, however as pattern of associations may differ by sex, it should be considered as a limitation that this was not included. A clear strength of preventing recall bias is the use of the KIDSCREEN-27 questionnaire administered in this study with only 1-week recall period [45] and that this current study had a relatively large sample size in a research field wherein large studies are scarce. Finally, the high variability of response rates across schools (from 8.6 to 92.1%) should be considered as a limitation. The response rate was lowest in two large schools in the east of Norway and highest in two small schools in the south of Norway, but due to the rules of General Data Protection Regulation laws, we were not allowed to collect information to assess how participants and nonparticipants, nor the respective schools differed.

### Clinical implications

In light of our current findings, it is of great importance to emphasize that sufficient sleep in schooldays among a school-based sample of adolescents seems to play a significant role of their perception of school environment. Given that the HRQOL subscale of school environment includes four questions related to the adolescents´ happiness in school, performance in school, concentration at school and relation to the teachers, employees at Norwegian high schools, including school nurses, teachers and school management should highlight and promote the importance of sufficient sleep among their pupils, especially during schooldays. Our findings indicate that interventions targeting enhancement of HRQOL school environment in adolescents, should strive to focus on increasing sleep duration.

Moreover, the Norwegian Ministry of Education and Research presented a revised and updated educational plan (LK20) for adolescents in this respective age-group in 2020, which included interdisciplinary subjects such as public health and life mastery subject. Despite “living habits” is mentioned in the description of learning outcomes in LK20, we advise practice and policy to be aware of the potential benefits of sleep duration in schooldays for Norwegian adolescents and advice to explicitly include information of sleep as a learning outcome for Norwegian adolescents. Thus, more extensive research evidence is needed to understand how sleep duration, quality and productivity affects HRQOL and how it relates to self-efficacy among a school-based sample of adolescents. Larger observational and longitudinal studies should strive for identifying relevant underlying mechanisms, the practical significance, effect sizes and provide solid recommendations for practice and policy.”

## Conclusions

This cross-sectional study described sleep among Norwegian adolescents and demonstrated that sleep durations in weekends and schooldays impact HRQOL and self-efficacy, revealing overall better outcome in HRQOL and self-efficacy with sufficient sleep during schooldays than sleep during weekends. Self-efficacy was found as a mediator on the relationship between sleep duration in schooldays and HRQOL subscales and thus provided insight in underlying mechanisms of the association. Revealing that sleep duration explained most of the associations in 4 out of 5 HRQOL subscales, except in the association with the HRQOL subscale physical well-being. These findings support the regularity of sleep and highlight the importance of sufficient sleep during the schooldays, especially in a school-based sample of adolescents.

## Data Availability

The datasets used and/or analyzed during the present study are not publicly available due to the General Data Protection Regulation laws but are available from the corresponding author on reasonable request and with permission from the Norwegian Centre for Research Data.

## Abbreviations

CI: confidence interval
HRQOL: health-related quality of life
SD: standard deviation
SOT: sleep onset time
